# Necrosis of the Inferior Maxillary

**Published:** 1851-07

**Authors:** 


### ARTICLE XIV.

### *Necrosis of the Inferior Maxillary.*

Mr. Stanley brought before the public of St. Bartholomew's Hospital, a patient who had lost the whole of his lower jaw by necrosis. The man, who looked about five or six and twenty, had been six months in the hospital, and traced the affection of the jaw to the effects of the vapors of phosphorus, to which his trade of lucifer-match maker had exposed him. The whole body of the lower maxilla, with one of the condyles, were exhibited, and presented several necrosed fragments, in which the original shape of the bone could easily be recognized. Mr. Stanley mentioned that the condyle, which was wanting to complete the necrosed jaw, had very probably been absorbed. The patient's appearance did not at first sight betoken such considerable osseous loss, and the usual contour of the face was tolerably preserved. This small amount of deformity was accounted for by the eminent surgeon under whose care he had been, by the thickening of the tissues in contact with the jaw, and an abundant fibrinous deposit which nature had thrown out, in her endeavor to repair the loss of bone which the patient had suffered. New bone, however, is never thrown out in this form of necrosis. Mastication, in this instance, is carried on tolerably well; the food must be reduced into small fragments, which the patient prepares further by pressing them strongly against the roof of the mouth with his tongue. The function of nutrition seems to be going on tolerably well with this patient, for he looks in pretty good health. It may then, be inferred, that this imperfect preparation of the alimentary bolus proves sufficient for the digestive functions. The painful feelings excited by the sight of this new victim of our vaunted improvements in various branches of manufacture were greatly mitigated by the announcement Mr. Stanley subsequently made, regarding the prophylaxis of these dreadful affections. Workmen in lucifer-match manufactories have now a chance of escaping the baneful effects of the evolution of phosphorous acid, by placing saucers filled with oil of turpentine about their workrooms. As oil of turpentine is a solvent of phosphorus, it is expected that it will absorb the vapors which do so much mischief. This precaution is taken at a large lucifer-match manufactory in the neighborhood of the London Hospital, and the very best results are expected from it. This case, so very interesting and instructive in itself, was rendered peculiarly valuable in being brought forward by the distinguished author of the recently published treatise on "Diseases of the Bones;" and we were sorry that the time and place did not allow Mr. Stanley to enter fully into the different questions which such a case will naturally give rise to; for instance, as to whether the fumes act primarily on the periosteum, or whether the jaw is secondarily affected, as a consequence of the contamination of the system. This question, which is left unsettled in the work just alluded to, appears of some importance; for if it were made tolerably clear that the constitution is affected first, the poison might be counteracted by throwing appropriate chemical agents into the blood. Indeed, we should be much inclined, from the analogy offered by the effects of mercury, to think that the vapors act but secondarily on the jaw, though it would appear that the system must be in a peculiar condition to become affected. The two cases mentioned in the last number of the Lancet, by Mr. Henry Taylor, of Nottingham, will prove very valuable to those who may investigate this subject in all its bearings ; for here we have two men, who were employed for a considerable time at the manufactory before they experienced any ill effects; whilst in one the upper, and the other the lower jaw, suffered necrosis. These men were "dippers;" and it is but fair to suppose that the hand would have been the first to suffer, had the effect been direct. It must, however, be confessed, that the fact of the *upper* maxilla being affected, in Case 1, militates against the following passage, which we find at p. 75 of Mr. Stanley's excellent treatise: "Against the opinion that the phosphoric vapor acts merely as a local excitant, the objection has been urged, that it produces no effect on the periosteum of the bones of the nasal passages through which the vapor is directly inhaled." That these cases should not be classed among ordinary necrosis of bones is sufficiently obvious, the entire absence of the least attempt at the regeneration of bone being the most characteristic differential feature. Nor could this regeneration well take place, as, to use Mr. Stanley's words, (p. 75,) "there is here a total want of the essential conditions for the reproduction of bone—namely, inflammation in healthy structures, with health in the general system." The grey, pumice-stone-like, newly formed osseous substance, found by Dr. Heyfelder on the outer surface of the portions of bone which he extracted in similar cases, likewise points, as Mr. Stanley remarks, to an affection distinct from the usual death of bone. We sincerely hope that the etiology of these affections will fix the attention of the many able investigators in surgical science of which this country can boast.

